# Metabolite-Induced Apoptosis by *Gundelia tournefortii* in A549 Lung Cancer Cells: A Cytotoxic and Gene Expression Study

**DOI:** 10.3390/nu17030374

**Published:** 2025-01-21

**Authors:** Aysun Yuksel, Damla Nur Celayir, Ezgi Nurdan Yenilmez Tunoglu, Lütfi Tutar, Yusuf Tutar

**Affiliations:** 1 Department of Nutrition and Dietetics, Medeniyet University, Istanbul 34720, Türkiye; aysun.yuksel@medeniyet.edu.tr; 2 Department of Nutrition and Dietetics, University of Health Sciences, Istanbul 34668, Türkiye; 3 Division of Medical Techniques and Services, Vocational School of Health Services, Demiroglu Science University, Istanbul 34394, Türkiye; ezgi.tunoglu@demiroglu.bilim.edu.tr; 4 Department of Molecular Biology and Genetics, Faculty of Arts and Sciences, Kırşehir Ahi Evran University, Kırşehir 40100, Türkiye; lutfi.tutar@ahievran.edu.tr; 5 Division of Biochemistry, Faculty of Pharmacy, University of Health Sciences, Istanbul 34668, Türkiye; 6 Division of Biochemistry, Faculty of Medicine, Recep Tayyip Erdogan University, Rize 53100, Türkiye

**Keywords:** *Tournefort’s gundelia* (kenger), *Gundelia tournefortii*, human lung carcinoma (A549), apoptosis, array

## Abstract

Background/Objectives: *Gundelia tournefortii* (Kenger) is a traditional medicinal plant and exhibits potential anticancer properties. This study investigates the cytotoxic and apoptotic effects of its water extract on human lung carcinoma A549 cells. Methods: A lung cancer cell line was treated with *Gundelia tournefortii* extract. The metabolic content of the extract that plays key roles in anticancer was detected by high-performance liquid chromatography. Anticancer properties were further detected by a flow cytometer apoptosis assay, and signaling pathways were determined by a PCR array through hub gene expression alteration. Gene enrichment analysis and network pharmacology correlated metabolites and pathways that were involved in anticancer effects. Results: The metabolite content of *G. tournefortii* was analyzed, and gallic acid, clorogenic acid, hydroxybenzoic acid, caffeic acid, epicatechin, p-coumaric acid, salicylic acid, apigenin 7 glucoside, and cinnamic acid were detected as key compounds. Lung cancer cell line A549 was treated with the extract at increasing concentrations for 24, 48, and 72 h, and its effects on cell viability were determined by MTT analysis. A statistically significant difference was observed for IC_50_ concentrations depending on incubation times. It was also observed that the *G. tournefortii* water extract significantly increased apoptosis in A549 cells in comparison with the control group. *G. tournefortii* extract’s effect on lung cancer cell line was measured using the signal pathway PCR array gene set. Gene enrichment analysis of the array expression data confirmed activation of apoptosis-related pathways, particularly the upregulation of BAX and downregulation of HSP90. Conclusions: These findings suggest that *G. tournefortii* metabolites provide promising selective anticancer drug candidates and potential drug templates to prevent side effects and resistance of current clinical drug treatments.

## 1. Introduction

Cancer is an important global health problem with its increasing incidence and mortality [1]. It occurs in association with DNA damage, but the reason why DNA damage occurs is not yet clearly known. However, it is known that together with inalterable factors such as hereditary conditions, age, and gender, along with alterable factors such as alcohol and cigarette consumption, air pollution, and unhealthy lifestyle, it has an impact on cancer formation and progression [2]. While there are more than a hundred cancer types affecting humans [3], lung cancer is the most common cancer type in Türkiye, at a rate of 17.6% [4]. The diagnosis and treatment of cancer require advanced medical technology and health expenditures [5]. Since these expenditures create a burden on social resources, efforts to prevent cancer are increasing day by day, and traditional treatments are becoming more popular.

About 85% of cases of lung cancer are non-small cell lung cancer (NSCLC), which continues to be the primary cause of cancer-related death worldwide [6]. Despite improvements in early detection and treatment, the 5-year survival rate for NSCLC is still only about 24%, highlighting the critical need for innovative therapeutic approaches (Cancer.org).

Conventional treatments, such as platinum-based chemotherapy, are linked to serious toxicity, like nephrotoxicity and neurotoxicity, and frequently show limited efficacy because of acquired resistance [7].

Natural products, which have long been a rich source of anticancer agents, such as vincristine and paclitaxel, have become the focus of the search for alternative therapies [8]. *Gundelia tournefortii* (Kenger) has drawn attention for its medicinal properties. *G. tournefortii*’s high phenolic content is attributed to its demonstration of anti-inflammatory, antioxidant, and potential anticancer activities.

Eating fruits and vegetables can reduce the risk of lung cancer by up to 18%, according to a recent systematic review. The high levels of flavonoids and other phenolic chemicals found in fruits and vegetables are thought to be the cause of the lower risk of lung cancer [9].

Several natural dietary compounds reported that phenolic compounds in particular display protective and therapeutic benefits against various forms of human cancers. These compounds with their functional groups activate several anticancer mechanisms and induce apoptosis, autophagy, cell cycle arrest, and suppress telomerase. Moreover, in vivo research indicates that these phenolic compounds suppress invasion and angiogenesis. Additionally, clinical research has already emphasized specific phenolic compounds that exhibit clinical effects either on their own or in combination with clinical chemotherapy drugs [10].

Many plants are used for therapeutic purposes in Anatolia. *G. tournefortii*, one such plant genus, is a perennial and thorny plant belonging to the family Asteraceae. While it blooms purple in March, April, and May, its head turns a yellowish green color in maturity [11]. *Gundelia tournefortii*, *Tournefort’s gundelia*, is known in Türkiye as “kenger”, “sweet kenger”, “kanak sakızı”, and “çadır dikeni” [12]. Regions of the Asian continent with moderate climates, particularly Türkiye, Iran, Cyprus, Egypt, Azerbaijan, Turkmenistan, Israel, and Jordan, are natural growing areas of *G. tournefortii* [13]. In Türkiye, it is most commonly seen in the eastern, central, southeastern, and Mediterranean regions [12]. In Southeast Anatolia and East Anatolia, *G. tournefortii* (“kenger”) is collected in spring and consumed as a vegetable after the stem is cleaned; in the Mediterranean and Central Anatolia regions, its fruits are dried, ground, and consumed as a beverage [14,15]. *G. tournefortii* gum is made from the milky latex released as a result of scratching the roots and stems of the plants with a knife [14]. The latex of *G. tournefortii* can be used as a stabilizer in ice cream production [16]. Roasted *G. tournefortii* with egg, *G. tournefortii* meal, *G. tournefortii* with olive oil, and *G. tournefortii* patties are among the dishes prepared with these plants [17,18,19]. In addition to its use as food, it is also used for therapeutic purposes in traditional medicine due to its rich phenolic compounds. In a previous study, it has been shown that *G. tournefortii* has anticancer, anti-inflammatory, antioxidant, anticholesterolemic, antibacterial, hepatoprotective, and hypoglycemic effects [20].

The plant is thought to have nutritional and therapeutic properties in folk medicine. The stem, leaves, roots, and fruits of the plant can be used to make an internal decoction that can be used to treat diabetes, epilepsy, fever, colds, coughs, renal pain, stomach, and intestinal conditions [21,22].

An external decoction of the latex has long been used to treat inflammation, toothaches, vitiligo, and edema. In Lebanon, latex is used as an emetic, to burn off warts, to dry up ulcers, and to prevent snake bites. It is also used as chewing gum because of its antibacterial qualities. According to studies, *G. tournefortii* contains antibacterial, hepatoprotective, antioxidant, and anticancer properties [21,22].

*G. tournefortii* includes six distinct phytochemicals with anticancer properties: lupeol, gitoxigenin, α-amyrin, artemisinin, sitosterol, and stigmasterol. *G. tournefortii*’s roots and disseminules are used to make Kenger herbal coffee, a coffee alternative that has drawn interest for its potential to treat oxidative stress-related illnesses like cancer and neurological diseases [21,22].

Phenolic compounds such as gallic acid, chlorogenic acid, and apigenin exhibit multiple anticancer mechanisms, such as induction of apoptosis, inhibition of angiogenesis, and modulation of critical signaling pathways like PI3K/Akt and MAPK [19,23]. Apoptosis plays a pivotal role in preventing the proliferation of damaged or cancerous cells. The intrinsic (mitochondrial) pathway, regulated by BCL2 family proteins, ultimately leads to cell death.

Recent findings have also focused on the critical role of heat shock proteins, mainly HSP90, in cancer cell survival. HSP90 functions as a molecular chaperone that regulates multiple oncogenic proteins, and, therefore, HSP90 has become a key target for cancer therapy. Once HSP90 function is disrupted, its client proteins are dictated to degrade, making cancer cells more susceptible to apoptosis [23].

Phenolic constituents of *G. tournefortii* are promising potential candidates for cancer treatment; however, limited studies have evidenced their effects on lung cancer cells. This study aimed to investigate the cytotoxic and apoptotic effects of *G. tournefortii* water extract on A549 lung cancer cells to identify the key phenolic compounds responsible and to elucidate the molecular mechanisms involved, including the modulation of apoptosis-related genes such as BAX, BCL2, CASP3, CASP9, and HSP90. Therefore, the present research was designed to examine the *G. tournefortii* metabolite effects against lung cancer at the molecular level.

## 2. Materials and Methods

This research was planned and carried out between February and December 2021 to determine the phenolic contents of a water extract obtained from stems of *G. tournefortii* by HPLC and to evaluate its potential effects on lung cancer. Approval of the study was obtained from the associated ethics committee (University of Health Sciences, Hamidiye Scientific Research Ethics Committee, registration number 21/485, dated 2 July 2021, numbered 23/5).

### 2.1. Source of Gundelia tournefortii and Preparation of Plant Extracts

Within the scope of this study, *G. tournefortii* was obtained from the city of Sanliurfa during harvest time. Plants were stored at −86 °C until used. At the start of the study, plant samples were moved to room temperature before preparation of the extract, and they were allowed to thaw. Thawed plants were ground using a grinder and turned into powder. Pure water was added to the powder as a solvent, and the extracts were boiled for one hour, filtered, and frozen at −20 °C. Extracts were subsequently placed in a lyophilizer, and the main drying process (sublimation) was undertaken for 16 h at 0.1 mBar pressure. After this stage, in order to remove the last water molecules that had been crystallized and adhered to the material, the ambient pressure was dropped to 0.01 mBar, and the final drying was carried out. Dried samples were turned into a powder using a pestle and preserved at −20 °C.

### 2.2. Determination of Phenolic Components

Determination of phenolic components was carried out by an HPLC-DAD system (Shimadzu Nexera-i LC-2040 3D Plus, Kyoto, Japan) with a C18 phenylhexyl column of 4.6 × 150 mm, 3 µm (UP). For the process of determination with gradient elution, 0.1% formic acid solution and acetonitrile were used as mobile phases. The mobile phases were filtered with a membrane filter of 0.45 µm, and air bubbles were removed. The injection volume was set to 10 µL, the flow rate was set to 1 mL/min, the temperature was set to 30 °C, and measurements were performed at a wavelength of 254 nm. Compounds were screened with a PDA detector. Gradient performed as time-min; mobile phase B (% acetonitrile); mobile phase A (% water/0.1% formic acid) as 0.01;5;95, 7;9.5;90.5, 20;17;83, 35;40;60, 40;0;100, 40.01 stop, respectively. Since *G. tournefortii* is edible, water extract was chosen to understand the metabolite effect on cancer wittingly (see Supplementary Material File). Control experiments were also performed to rule out any artifact effects.

### 2.3. Cell Line Culture Studies

To determine the anticancer activity of *G. tournefortii*, a human lung cancer cell line (A549) was used. A549 cells were cultivated in the DMEM high glucose growth medium supplemented with 10% FBS and a mixture of 1% penicillin/streptomycin. Cells collected previously and diluted in medium were seeded into 96-well plates with 100 µL of 1 × 10^4^ cells in each well. These seeded cells adhered to the plate’s surface within 24 h. The next day, extracts with predetermined concentrations (100 µg/mL, 50 µg/mL, 25 µg/mL, 12.5 µg/mL, 6.25 µg/mL, 3.125 µg/mL, and 1 µg/mL) were left to incubate for 24, 48, and 72 h.

### 2.4. Determination of Cell Viability

Commercially obtained MTT was used for cell viability experiments, containing 3-(4,5-dimethylthiazol-2-yl)-2,5-diphenyltetrazolium bromide. The MTT solution was prepared by mixing 5 mg/mL of MTT agent in PBS. MTT is a tetrazolium salt that is decomposed by the dehydrogenase enzyme in the mitochondria of metabolically active cells and turns into water-soluble formazan. The intensity of the purple color arising from formazan is proportionate to the number of living cells. Sterile 96-well plates were seeded with 1.0 × 10^4^ cells in each well, and the next day, the medium in each well was carefully aspirated to determine the viability of the extract-treated cells. Medium (90 µL) and MTT solution (10 µL) were added to each well and left in the incubator for 3–4 h. At the end of this time, the plates were incubated at 37 °C in a shaking incubator for 15 min in the dark. Later, 100 µL of DMSO was added, and the color intensity was measured spectrophotometrically with an ELISA reader device (BioTek, Synergy Neo2, Agilent, Winooski, VT, USA) in the wavelength range of 570 nm. Each evaluation was compared with the number of cells in the positive control group at the same time, and the percentages of cytotoxicity were calculated using the following formula: Cytotoxicity % = 1 − [(Mean of Absorbance of Treated Wells/Mean of Absorbance of Positive Control Wells) × 100]. As a result of MTT experiments, IC50 values were calculated with the GraphPad program to determine the concentrations at which 50% cell death occurred.

### 2.5. Array Experimental Method

To understand the effect of the extract on cancer pathways, PCR array studies were employed to screen the biochemical pathways involved due to *G. tournefortii*. The array displays the effect in terms of cancer signaling pathways, cell cycle, cell death, and transcriptional regulation of hub genes. Cells were cultured at 1 × 10^6^ cells/well in 6-well plates for 48 h at 37 °C and 5% CO_2_. The cells were treated with *G. tournefortii* extracts at IC_50_ concentrations (48 h) and compared to those of the untreated A549 cancer cell line using the gene expression differences in the array. The Trizol reagent (Invitrogen, Waltham, MA, USA) was used to extract total RNA, following the instructions. High-quality RNA samples were then reverse-transcribed to obtain cDNA. cDNA was then subjected to PCR. SYBR Green Real-Time PCR Master Mix (SensiFAST, Bioline, Meridian Bioscience, OH, USA) was used to prepare PCR reactions. The following parameters were employed for the PCR reactions: an initial denaturation step at 95 °C for 5 min, followed by 40 cycles of 15 s at 95 °C and 30 s at 60 °C. The data were then normalized using the 2^−ΔΔCT^ method. Then, by using the cancer array gene set primers (ordered from Merck, and the primers were HPLC purified), RT-pPCR results were examined with gene enrichment analysis with previously established protocols in our lab [24,25,26]. Gene enrichment analysis indicates that the *G. tournefortii* extracts drive A549 cancer cells to apoptosis and arrest the cell cycle. Therefore, our lab performed flow cytometry experiments to support these outcomes [24,25,26].

### 2.6. Determination of Apoptosis with Flow Cytometry

Apoptosis analysis was carried out with an annexin V-FITC kit (Merck Millipore, Darmstadt, Germany). Annexin V-FITC facilitates the fluorescent detection of annexin V bound to apoptotic cells and its quantitative determination by flow cytometry (CytoFLEX, Beckman Coulter, CA, USA). In the annexin V-FITC kit, annexin V conjugated with FITC is used to label the phosphatidylserine sites on the membrane surface. The kit contains PI to label cellular DNA in necrotic cells in which the cellular membrane is completely compromised. This combination allows for differentiation between early apoptotic cells (annexin V-positive, PI-negative), necrotic cells (annexin V-positive, PI-positive), and viable cells (annexin V-negative, PI-negative).

For this staining method, 1 × 10^6^ cells were first cultured in 6-well cell plates and left to incubate for 24 h at 37 °C in 5% CO_2_. At the end of 24 h, *G. tournefortii* extract at the IC_50_ concentration was added to one of the wells, and incubation was continued for 48 h. After this treatment, at the end of the 48th hour, the medium of all wells, both with and without extract application, was removed, and 0.25% trypsin was added to the wells, which were washed with PBS and incubated at 37 °C for 2–3 min in an environment with 5% CO_2_. The contents of all wells were transferred to individual 15 mL centrifuge tubes and centrifuged at 130× *g* for 5 min, checking that the cells were fully removed from the surface under a microscope. After these procedures, the protocol as suggested by the kit’s manufacturer was applied, and analysis was performed with a flow cytometer device (Beckman Coulter CytoFLEX, Brea, CA, USA) with gentle vortexing [24,25,26].

### 2.7. Network Pharmacology

Traditional Chinese Medicine Systems Pharmacology Database and Analysis Platform (TCMSP, https://old.tcmsp-e.com/tcmspsearch.php) (accessed on 27 November 2024) was used to determine metabolite–gene relationships. Metabolite cancer and cancer gene targets were determined by TCMSP by employing array analysis. Determined gene targets were employed in enrichment analysis, and the analysis, along with metabolites from HPLC analysis, revealed that *G. tournefortii* metabolites drive lung cancer cells to apoptosis. Further metabolite composition of *G. tournefortii* extracted from a recent study [27] employed in TCMSP and the human metabolome database (https://hmdb.ca) (accessed on 27 November 2024) also compared to differentially expressed genes of the current study, and network pharmacology results supported the same result; *G. tournefortii* extract drives the A549 cancer cell line to apoptosis [28].

### 2.8. Statistical Analysis

For the statistical analysis of the data obtained in this research, GraphPad Prism 7.0 and IBM SPSS Statistics 26.0 were used. Nonlinear logistic regression analysis (4PL) was used for evaluation of MTT assays, and chi-square analysis was applied for flow cytometry evaluations. Statistical significance was accepted at *p* < 0.05.

## 3. Results

### 3.1. HPLC Analysis Findings

HPLC analysis detected 4.638 mg/L chlorogenic acid, 1.886 mg/L apigenin-7-glucoside, 0.372 mg/L gallic acid, 0.185 mg/L chicoric acid, 0.100 mg/L p-coumaric acid, and 0.012 mg/L cinnamic acid in the water extract of *G. tournefortii* (chromatograms are given in the Supplementary Material File).

### 3.2. Anticancer Activity of Gundelia tournefortii Extract

**Figure 1:**
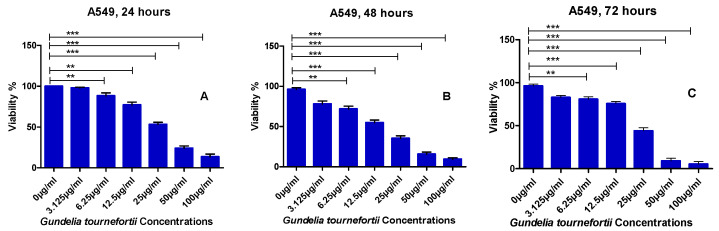
Percent cell viability of the A549 cell line for 24 h (**A**), 48 h (**B**), and 72 h (**C**) at increasing concentrations of *G. tournefortii* water extract (**: *p* < 0.01, ***: *p* < 0.001).

Graphs of cell viability (%) obtained with increasing concentrations of the *G. tournefortii* water extract (1 µg/mL, 3.125 µg/mL, 6.25 µg/mL, 12.5 µg/mL, 25 µg/mL, 50 µg/mL, 100 µg/mL) incubated with A549 cells for 24, 48, and 72 h are given in Figure 1. Dose-dependent decrease of A549 cell viability was observed as a result of *G. tournefortii* extract application.

**Figure 2:**
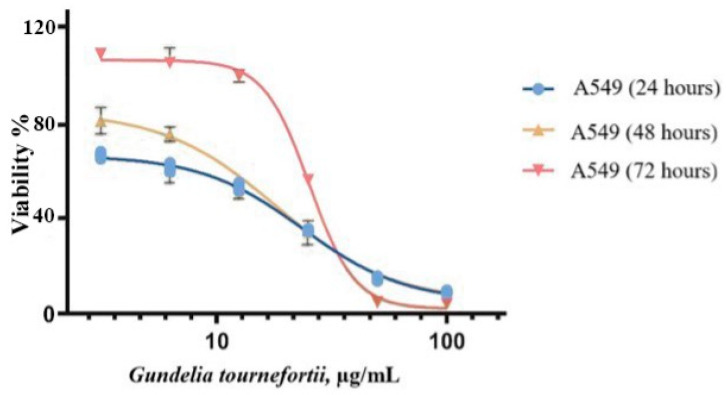
IC_50_ values of *Gundelia tournefortii* extracts against A549 cells with error bars. Statistical significances are *p*: 0.01, *p* < 0.01, and *p*: 0.01 for 24, 48, and 72 h, respectively.

The dose-dependent effects of the *G. tournefortii* extract on the viability of A549 cells and the concentrations (IC_50_) that provided 50% inhibition of A549 cells at 24, 48, and 72 h are given in Figure 2.

The IC_50_ values of *G. tournefortii* extract against A549 cells at 24, 48, and 72 h were calculated as 23.6 ± 0.3 µg/mL at 24 h, 18.2 ± 0.4 µg/mL at 48 h, and 25.2 ± 0.6 µg/mL at 72 h (Figure 2). R^2^ values were found to be high and appropriate for model compliance in all groups. An increase was observed in peak slope and span as the application time increased. Furthermore, a statistically significant difference was detected in IC_50_ concentrations according to incubation times (*p* < 0.0001). The cell viability experiments indicated potential anticancer effects of the metabolites, but to understand the cancer pathways and biochemical processes involved at the molecular level, array experiments were performed.

### 3.3. Array Data Analysis by Gene Enrichment Method

**Figure 3:**
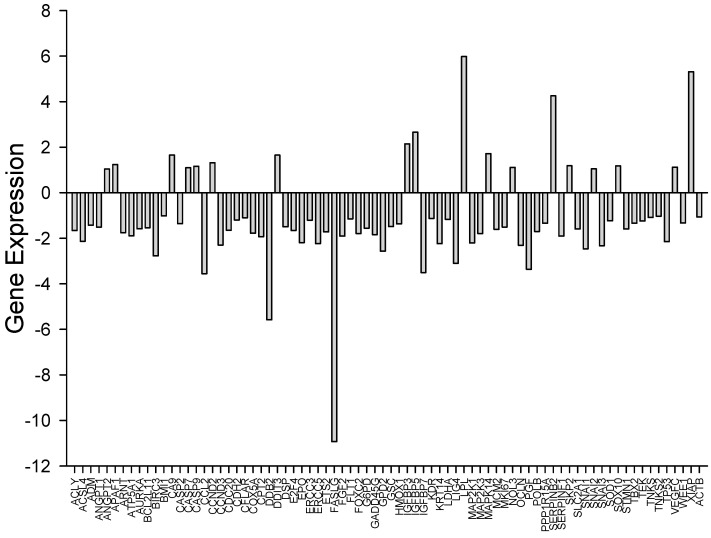
Expression differences of cancer array genes in the presence of *Gundelia* extract in A549 cells. Hub gene expression enhancements provide elucidation of pathways provided in Table 1 and the clustergram in Figure 4.

**Figure 4:**
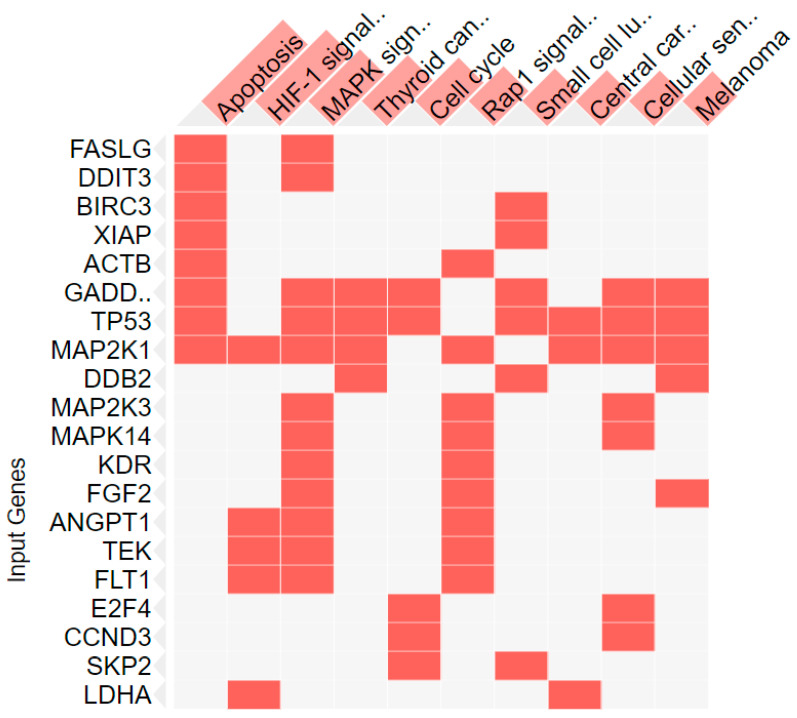
Clustergram of the Enrich tool. Gene enrichment analysis of cancer array genes in the presence of *G. tournefortii* extract determined differentially expressed hub genes and key pathways related to these genes displayed. Pathways in this figure are given in Table 1 with statistics.

Expression differences of hub genes in cancer arrays indicate that *G. tournefortii* extract displays anticancer activity against A549 (Table 1 and Table 2 and Figure 3 and Figure 4), as revealed by gene enrichment analysis through EnrichR software (https://maayanlab.cloud/Enrichr/) (accessed on 27 November 2024) [24,25,26,29,30,31]. Gene enrichment analysis indicates that the extract metabolites drive cancer cells to apoptosis, and flow cytometry experiment results support this outcome. The enrichment analysis also indicates that the extract is an effective anticancer agent. Table 1 and Figure 4 indicate that the metabolites drive cancer cells to apoptosis and arrest the cell cycle by employing multiple biochemical pathways, including MAPK, PI3K-Akt, and HIF-1 signaling. It is not surprising that these pathways are involved in cell survival, proliferation, and apoptosis.

### 3.4. Determination of Apoptosis

**Figure 5:**
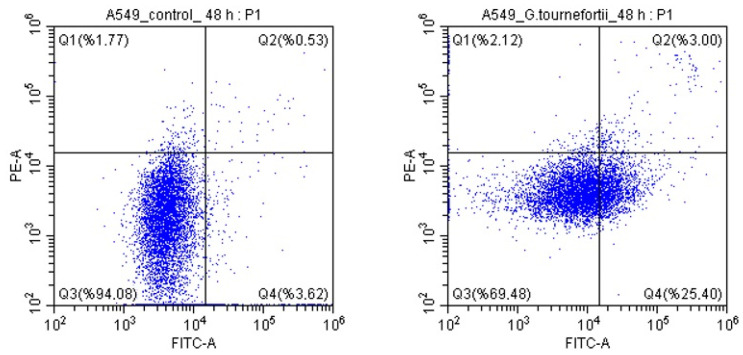
Flow cytometry analysis of *G. tournefortii*-treated cells and control group cells apoptotic rates. (**Left**) control; (**right**) *G. tournefortii*-treated cells. Percentages are given in Table 2.

Flow cytometry analysis (%) values of the cells of the control group and the cells of the experimental group to which *G. tournefortii* extract was administered are given in Table 2. When the *G. tournefortii* group was compared to the control group, a statistically significant increase was observed in the number of cells displaying late apoptosis and early apoptosis, and a statistically significant decrease was also observed in the number of viable cells (*p* < 0.0001). The results (Q2 + Q4) indicate that the extract drives A549 lung cancer cells to apoptosis (Figure 5).

### 3.5. Network Pharmacology Analysis

*G. tournefortii* metabolites determined by HPLC and hub genes on cancer pathways from PCR Array were correlated. The metabolites of *G. tournefortii* extract drive A549 cells to apoptosis through BCL2, BAX, CASP9, CASP3, and HSP90 (Table 3).

The analysis also revealed that the immune system (both innate and cytokine signaling) was induced by HSP90, BCL2, IGHG1, MAOA, COX2, CASP3, NOS1, GLB1, PTPN1, CTSD, PRSS3, SRC, CASP9, and LYZ genes.

To further support our results with literature reports, metabolite composition of *G. tournefortii* from a recent study [23] screened in TCMSP and in the human metabolome database (https://hmdb.ca) (accessed on 27 November 2024). Network pharmacology outcomes of this data supported our current research results.

## 4. Discussion

As each day passes, cancer cases are increasing and becoming an important problem for the health of all societies. Cancer is a disease that is expensive both to diagnose and treat, and it causes various side effects. That is why the importance of herbal products for the prevention and treatment of cancer is also steadily increasing.

*G. tournefortii* is a plant rich in phenolic contents. In a study carried out by Abu-Lafi et al. [32], when the phenolic contents of water, methanol, and hexane extracts of *G. tournefortii* collected in March in Palestine were studied using GC-MS analysis, stigmasterol, β-sitosterol, methyl palmitate, lupeol, ethyl oleate, α-amyrin, palmitic acid, linoleic acid, myristic acid, gitoxigenin, and artemisinin compounds were detected [32]. In another study [11], seventy different compounds were found when *G. tournefortii* plants collected from the Zagros region of Iran were studied with GM-MS analysis. It was reported that the basic components were palmitic acid (12.48%), lauric acid (10.59%), alpha-ionene (6.68%), myristic acid (4.45%), 1-hexadecanol-2-methyl (3.61%), phytol (3.6%), and beta-turmerone (3.4%). In another study, when the phenolic contents of *G. tournefortii* plants growing in the Upper Euphrates Basin were examined by HPLC analysis, 0.5–22.5 mg/kg quercetin, 10.86–21.8 mg/kg kaempferol, 1.25–7.5 mg/kg naringenin, 1.25–6.75 mg/kg myricetin, and 1.25–3.75 mg/kg resveratrol were detected [33]. In a study carried out in Türkiye, when the phenolic contents of the ethanol extract of *G. tournefortii* root and stem were examined by GC-MS analysis, 22 different compounds were detected, the ones with the highest levels being palmitic acid, lauric acid, myristic acid, 1-hexadecanol, 2-methyl, octadecane, and heneicosane [34]. Leaf samples obtained during and after the flowering period of *G. tournefortii* and the caffeic acid derivatives in the seeds were studied, 984, 466, and 199 mg of chlorogenic acid were detected, respectively in 100 g of dried plant [35]. In this study, when the phenolic contents of *G. tournefortii* collected in Şanlıurfa were examined by HPLC analysis, gallic acid, p-coumaric acid, chicoric acid, apigenin-7-glucoside, chlorogenic acid, and cinnamic acid were found.

*G. tournefortii* is known to have antioxidant, anti-inflammatory, anticancer, antibacterial, hepatoprotective, hypoglycemic, and hypocholesterolemic effects, as it is rich in phenolic compounds [20,36,37]. *G. tournefortii* decreased the viability of HCT-116 human colon cancer cells when a methanol and hexane extract of *G. tournefortii* was applied. It has been reported that this anticancer activity stems from the compounds of stigmasterol, β-sitosterol, lupeol, α-amyrin, gitoxigenin, and artemisinin [36]. In another study, a moderate antiproliferative effect of the water extract of *G. tournefortii* seeds on human prostate cancer (PC-3), human gastric cancer (MKN-45), human breast cancer (MCF-7), and human endothelial (HUVEC) cell lines was identified, and it was also found to have a weak antiproliferative effect on a mouse fibroblast cell line (L929) [37]. In another study in which the water extract of *G. tournefortii* was used, a cytotoxic effect was observed for both the MCF-7 and HUVEC cell lines [38]. In this study as well, it was found that the application of increasing concentrations of the water extract of *G. tournefortii* exerts cytotoxic effects against the A549 human lung cancer cell line. In light of all of these results, *G. tournefortii* demonstrates cytotoxic effects against many types of cancer.

Also, IC_50_ levels of the water extract of *G. tournefortii* seeds collected in Sivas, a province of Türkiye, were examined for MKN-45, PC3, MCF-7, HUVEC, and L929 cell lines after 24 h of incubation, and the lowest IC_50_ value was detected for the PC3 cell line (68.855 μg/mL) [37]. IC_50_ values were found to be 237.4 μg/mL for the MKN-45 cell line, 348.5 μg/mL for the MCF-7 cell line, 458.3 μg/mL for the L929 cell line, and 612.2 μg/mL for the HUVEC cell line.

In the present study, the IC_50_ values achieved against a human lung cancer cell line after 24, 48, and 72 h of incubation with *G. tournefortii* water extract were 23.6 ± 0.3 µg/mL, 18.2 ± 0.4 µg/mL, and 25.2 ± 0.6 µg/mL, respectively. Significant differences were noted among these IC_50_ values depending on incubation time. For this reason, it is thought that it is necessary to adjust the *G. tournefortii* dose according to the administration time. Different cancer types and the use of different parts of the plant are thought to be the reasons for the differences in IC_50_ levels [37].

In another report [39], examining the effects of *G. tournefortii* on mouse hepatocellular carcinoma, it was found that 47.03% of the control group cells underwent early apoptosis, and, as a result of treatment with 50, 100, and 200 µg/mL *G. tournefortii* extract, the rates of apoptotic activity increased to 55.67 ± 4.4%, 53.67 ± 4.4%, and 62.67 ± 7.3%, respectively (*p* < 0.02). A significant decrease in necrosis was also observed (*p* < 0.004), and it was reported that *G. tournefortii* showed anticancer properties by programming the death of hepatocellular cancer cells. In the present study, when A549 lung cancer cells were incubated with the IC_50_ concentration of *G. tournefortii* extract (18.2 ± 0.4 µg/mL) for 48 h, the rates of early apoptosis, late apoptosis, and necrosis increased while the percentage of viable cells decreased. While the increase in the rate of apoptosis and the decrease in the rate of viable cells were found to be significant (*p* < 0.0001), the increase in necrosis was statistically insignificant (*p* = 0.209).

The current study employed the metabolic effect of the *G. tournefortii* over a lung cancer cell line. Differentially expressed genes by *G. tournefortii* imply that the phenolic content drives the cancer cells to apoptosis as revealed by gene enrichment analysis. Gene enrichment analysis also revealed cell cycle arrest of the cancer cell line.

The results indicate that the phenolic content affects the immune system. This is the main target for cancer to suppress and capture the whole molecular system in a cell. Furthermore, HSP90 is likely suppressed since HSP90 has an anti-apoptotic and functional role in the immune system. Cancer uses the HSP system to enhance metabolism for tumoral needs. HSP system is a survival system, and most HSP isoforms’ expressions are increased in cancer. They are also involved in cell cycle protein folding processes. Therefore, metabolites of *G. tournefortii* directly or indirectly affect the HSP system to drive cancer cells to apoptosis.

## 5. Conclusions

Within the scope of this study, the phenolic contents and potential cytotoxic and apoptotic effects of *G. tournefortii* plant samples collected from Şanlıurfa were investigated. According to the results obtained, the water extract of *G. tournefortii* stems contains gallic acid, chlorogenic acid, p-coumaric acid, chicoric acid, apigenin-7-glucoside, and cinnamic acid, and the administration of *G. tournefortii* water extract to the A549 lung cancer cell line provides a decrease in cancer cell viability. It was also observed that the *G. tournefortii* water extract induced apoptosis in this lung cancer cell line.

This study shows the prominent anticancer activity of *G. tournefortii* water extract on A549 lung cancer cells. The extract causes apoptosis mainly through mitochondrial pathways and has cytotoxic effects due to its high phenolic content. Foremost phenolics like gallic acid and chlorogenic acid interfere with cancer cell metabolism. On the other hand, gene expression analysis confirms the modulation of critical apoptotic regulators such as BAX, CASP3, and HSP90.

According to the results, *G. tournefortii* shows promise as an adjuvant cancer treatment. It is positioned as a possible adjunct to traditional therapies like chemotherapy due to its capacity to target proteostasis and trigger apoptosis. To confirm these findings and investigate the potential benefits of integrating *G. tournefortii* extracts with current treatment plans, this study becomes a background for further in vivo research and clinical trials.

This study provides evidence that *G. tournefortii* has potential anticancer activity and shows molecular pathways involved in the process. The reported anticancer activity of *G. tournefortii* with distinct cancer cell lines most likely occurred through the same apoptotic pathway. Thus, the metabolites provide promising anticancer drug candidates and potential drug templates to prevent side effects and resistance of current clinical drug treatments.

## Figures and Tables

**Table 1:** *G. tournefortii* extract enhancing A549 cell pathways as evidenced by gene enrichment analysis.

Index	Name	*p*-Value	Adjusted *p*-Value	Odds Ratio	Combined Score
1	Apoptosis	1.01 × 10^−9^	1.89 × 10^−7^	30.80	850.69
2	Pathways in cancer	9.51 × 10^−9^	5.97 × 10^−7^	12.31	312.49
3	MAPK signaling pathway	9.52 × 10^−9^	5.97 × 10^−7^	17.49	443.75
4	HIF-1 signaling pathway	2.34 × 10^−6^	1.10 × 10^−4^	27.56	547.74
5	Cell cycle	6.53 × 10^−6^	2.45 × 10^−4^	23.98	451.95
6	PI3K-Akt signaling pathway	1.74 × 10^−5^	5.46 × 10^−4^	11.64	207.87
7	Rap1 signaling pathway	2.51 × 10^−5^	6.74 × 10^−4^	15.78	276.13
8	Cellular senescence	3.96 × 10^−5^	9.31 × 10^−4^	18.76	319.82
9	Transcriptional misregulation in cancer	1.97 × 10^−4^	0.000004117	15.07	232.61
10	Focal adhesion	2.80 × 10^−4^	0.000004965	14.36	216.63

**Table 2:** Flow cytometry analysis (%) values of *G. tournefortii*-treated cells and control group cells.

	Control (%)	*Gundelia*(%)	*p* Value	Chi-Square
Q1	2 ± 1	2 ± 1	0.209	1.58
Q2	1 ± 1	3 ± 1	<0.0001	128.11
Q3	94 ± 2	70 ± 2	<0.0001	1412.79
Q4	4 ± 2	25 ± 2	<0.0001	1343.15
Q2 + Q4	5	28	<0.0001	1500.88

Q1: necrosis; Q2: late apoptosis; Q3: viable cells; Q4: early apoptosis; Q2 + Q4: early and late apoptosis.

**Table 3:** Gene enrichment analysis of A549 lung cancer cells treated with *G. tournefortii* extracts.

Index	Name	*p*-Value	Adjusted *p*-Value	Odds Ratio	Combined Score
1	Apoptotic signaling in response to DNA damage homo sapiens h chemical pathway	1.353 × 10^−8^	9.740 × 10^−7^	221.67	4016.27
2	Role of mitochondria in apoptotic signaling homo sapiens h mitochondrial pathway	0.000002090	0.00006376	161.76	2115.50
3	Stress induction of HSP regulation homo sapiens h hsp27 pathway	0.000002657	0.00006376	147.04	1887.82
4	Regulation of cell cycle progression by Plk3 homo sapiens h plk3 pathway	0.000005922	0.0001066	107.81	1297.69
5	Roles of beta-arrestin-dependent recruitment of Src kinases in GPCR signaling homo sapiens h b arrestin-src pathway	0.00004248	0.0006117	52.12	524.71

## Data Availability

All data generated or analysed during this study are included in this published article. The original datasets can be made available upon reasonable request to the corresponding author.

## Supplementary Materials

The following supporting information can be downloaded at https://www.mdpi.com/article/10.3390/nu17030374/s1, HPLC Chromatograms of Metabolite Analysis. Figure S1: Gallic acid metabolite analysis; Figure S2: Clorogenic acid metabolite analysis; Figure S3: p-coumaric acid metabolite analysis; Figure S4: Chicoric acid metabolite analysis; Figure S5: Apigenin-7-glucoside metabolite analysis; Figure S6: Cinnamic acid metabolite analysis; Figure S7: Overlay of standard chromatogram and sample chromatogram; Table S1: Gallic acid peak properties; Table S2: Clorogenic acid peak properties; Table S3: p-coumaric acid peak properties; Table S4: Chicoric acid peak properties; Table S5: Apigenin-7-glucoside peak properties; Table S6: Cinnamic acid peak properties; Table S7: Metabolic content of *G. tournefortii*.

## References

[1] Upadhyay A. (2020). Cancer: An unknown territory; rethinking before going ahead. Genes Dis..

[2] T.C. Ministry of Health, Department of Cancer (2018). Causes of Cancer and Common Cancers. https://tektiklabilgielinde.saglik.gov.tr/kanserin-nedenleri-ve-sik-gorulen-kanserler.html.

[3] Pavlopoulou A., Spandidos D.A., Michalopoulos I. (2015). Human cancer databases. Oncol. Rep..

[4] WHO Global Cancer Observatory: Cancer Today. https://gco.iarc.fr.

[5] Turkish Immuno-Oncology Society, Turkish Lung Cancer Society, Turkish Society of Medical Oncology and Turkish Thoracic Society. https://toraks.org.tr/site/sf/nmf/pre_migration/81b06b2de39bb1a1673bc0eb3b8394de4eeeb0bf21cc67264b7485e224d08566.pdf.

[6] Siegel R.L., Miller K.D., Jemal A. (2020). Cancer statistics. CA Cancer J. Clin..

[7] Herbst R.S., Morgensztern D., Boshoff C. (2018). The biology and management of non-small cell lung cancer. Nature.

[8] Newman D.J., Cragg G.M. (2020). Natural products as sources of new drugs over the nearly four decades from 1981 to 2019. J. Nat. Prod..

[9] Muller A.G., Sarker S.D., Saleem I.Y., Hutcheon G.A. (2019). Delivery of natural phenolic compounds for the potential treatment of lung cancer. Daru.

[10] Bakrim S., Omari N.E., Hachlafi E.N., Bakri Y., Lee L.H., Bouyahya B. (2022). Dietary Phenolic Compounds as Anticancer Natural Drugs: Recent Update on Molecular Mechanisms and Clinical Trials. Foods.

[11] Ceylan S., Cetin S., Camadan Y., Saral O., Ozsen O., Tutus A. (2019). Antibacterial and antioxidant activities of traditional medicinal plants from the Erzurum region of Turkey. Ir. J. Med. Sci..

[12] Anik R. (2019). Akbaldir (*Ornithogalum narbonense* L.) Also Used as a Food Product in Şanliurfa and Kenger (*Gundelia tournefortii* L.) the Effect of the Most Widely Used Cooking Methods of Plants on Phenolic Compound, Vitamin C Amount and Antioxidant Activity Values. Master’s Thesis.

[13] Farhang H.R., Vahabi M.R., Allafchian A.R. (2016). Chemical compositions of the essential oil of *Gundelia tournefortii* L. (Asteraceae) from Central Zagros, Iran. J. Med. Herbs.

[14] Konak M., Ateș M., Șahan Y. (2017). Evaluation of antioxidant properties of *Gundelia tournefortii*: A wild edible plant. J. Agric. Fac. Uludag Univ..

[15] Sirri M., Sirri G. (2020). Current Status of Wild Plant and Weed Species Consumed as Food in Hakkari Province. Eur. J. Sci. Technol..

[16] Cakmakci S., Dagdemir E. (2013). A preliminary study on functionality of *Gundelia tournefortii* L. as a new stabiliser in ice cream production. Int. J. Dairy Technol..

[17] Lezzetler, Gundelia Meal. https://lezzetler.com/tarif-67136.html.

[18] Yemektarifi, Gundelia with Olive Oil. https://1001yemektarifi.com/zeytinyagli-kenger-yemegi/.

[19] Nefisyemektarifleri, Roasted Gundelia with Egg. https://www.nefisyemektarifleri.com/yumurtali-kenger-kavurmasi/.

[20] Asadi-Samani M., Rafieian-Kopaei M., Azimi N. (2013). Gundelia: A systematic review of medicinal and molecular perspective. Pak. J. Biol. Sci..

[21] Hani N., Abulaila K., Howes M.J., Mattana E., Bacci S., Sleem K., Sarkis L., Eddine N.S., Baydoun S., Apostolides N.A. (2024). *Gundelia tournefortii* L. (Akkoub): A review of a valuable wild vegetable from Eastern Mediterranean. Genet. Resour. Crop Evol..

[22] Gezici S., Sekeroglu N. (2021). Comparative biological analyses on kenger and kenger coffee as novel functional food products. J. Food Sci. Technol..

[23] Ozgur. A., Tutar Y., Ozgur. A., Tutar Y. (2016). Heat Shock Protein 90 Inhibition in Cancer Drug Discovery: From Chemistry to Futural Clinical Applications. Anticancer Agents Med. Chem..

[24] Gümüş M., Koca İ., Sert Y., Dişli A., Tunoğlu E.N.Y., Tutar L., Tutar Y. (2023). Triad pyrazole-thiazole-coumarin heterocyclic core effectively inhibit HSP and drive cancer cells to apoptosis. J. Biomol. Struct. Dyn..

[25] Tunoğlu S., Tutar L., Gümüş M., Tunoğlu E.N.Y., Koca İ., Tutar Y. (2024). Hsp Inhibitor is Affective Against Adenocarcinomic Human Alveolar Basal Epithelial Cells Through Modulating ERK/MAPK Signaling Pathway. Chem. Biodivers..

[26] Çapan I., Hawash M., Qaoud M.T., Gülüm L., Tunoglu E.N.Y., Çifci K.U., Çevrimli B.S., Sert Y., Servi S., Koca I. (2024). Synthesis of novel carbazole hydrazine-carbothioamide scaffold as potent antioxidant, anticancer and antimicrobial agents. BMC Chem..

[27] Kadan S., Melamed S., Benvalid S., Tietel Z., Sasson Y., Zaid H. (2021). *Gundelia tournefortii*: Fractionation, Chemical Composition and GLUT4 Translocation Enhancement in Muscle Cell Line. Molecules.

[28] Tutar Y. (2024). Metabologenomics and network pharmacology to understand the molecular mechanism of cancer research. World J. Clin. Cases.

[29] Chen E.Y., Tan C.M., Kou Y., Duan Q., Wang Z., Meirelles G.V., Clark N.R., Ma’ayan A. (2013). Enrichr: Interactive and collaborative HTML5 gene list enrichment analysis tool. BMC Bioinform..

[30] Kuleshov M.V., Jones M.R., Rouillard A.D., Fernandez N.F., Duan Q., Wang Z., Koplev S., Jenkins S.L., Jagodnik K.M., Lachmann A. (2016). Enrichr: A comprehensive gene set enrichment analysis web server 2016 update. Nucleic Acids Res..

[31] Xie Z., Bailey A., Kuleshov M.V., Clarke D.J.B., Evangelista J.E., Jenkins S.L., Lachmann A., Wojciechowicz M.L., Kropiwnicki E., Jagodnik K.M. (2021). Gene set knowledge discovery with Enrichr. Curr. Protoc..

[32] Abu-Lafi S., Rayan B., Kadan S., Abu-Lafi M., Rayan A. (2019). Anticancer activity and phytochemical composition of wild *Gundelia tournefortii*. Oncol. Lett..

[33] Yildiz S. (2014). Determination of Phenolic Compounds and Metals in the Kenger (*Gundelia tournefortii* L.), Gulluk (*Eremurus tpectabilis* M. Bieb.) and Iskin (*Rheum ribes* L.) of the Plant Grown in Upper Euphrates Basin. Master’s Thesis.

[34] Keskin M., Kaya G., Keskin S. (2021). *Gundelia Tournefortii* L. (Kenger): Determination of in vitro Antidiabetic Activities. Prog. Nutr..

[35] Haghi G., Hatami A., Arshi R. (2011). Distribution of caffeic acid derivatives in *Gundelia tournefortii* L.. Food Chem..

[36] Hajizadeh-Sharafabad F., Alizadeh M., Mohammadzadeh M.H.S., Alizadeh-Salteh S., Kheirouri S. (2016). Effect of *Gundelia tournefortii* L. extract on lipid profile and TAC in patients with coronary artery disease: A double-blind randomized placebo controlled clinical trial. J. Herb. Med..

[37] Sarac H., Demirbas A., Dastan S.D., Atas M., Cevik Ö., Eruygur N. (2019). Evaluation of nutrients and biological activities of Kenger (*Gundellia tournefortii* L.) seeds cultivated in Sivas province. Turk. J. Agric.-Food Sci. Technol..

[38] Ozaltun B., Dastan T. (2019). Evaluation of antimicrobial activities and in vitro cytotoxic activities of *Gundelia tournefortii* L. Plant extracts. SDÜ Tıp Fakültesi Derg..

[39] Amer J., Jaradat N., Aburas H., Hattab S., Abdallah S. (2022). Gundelia Tournefortii extracts inhibit progressions of Hepatocellular Carcinoma in mice model through decrease in p53/Akt/PI3K signaling pathway. Biomed. Pharmacother..

