# Supplementary material for: Metabolite-Induced Apoptosis by Gundelia tournefortii in A549 Lung Cancer Cells: A Cytotoxic and Gene Expression Study
Source: Nutrients. 2025 Jan 21;17(3):374. doi: 10.3390/nu17030374 (PMC11820080; doi:10.3390/nu17030374)
Supplement: Supplementary file 1 [file nutrients-17-00374-s001.zip › nutrients-3385165-supplementary.pdf]

Metabolite Induced Apoptosis by *Gundelia tournefortii* in A549 Lung Cancer Cells: A Cytotoxic and Gene Expression Study

Aysun Yuksel[1], Damla Nur Celayir[2], Ezgi Nurdan Yenilmez Tunoglu[3], Lutfi Tutar[4], Yusuf Tutar[3,5,6,*]
[1]Medeniyet University, Department of Nutrition and Dietetics, Istanbul, Türkiye, [2]University of Health Sciences, Department of Nutrition and Dietetics, Istanbul, Türkiye, [3]University of Health Sciences, Health Sciences Institutes, Division of Molecular Medicine, Istanbul, Türkiye, [4]Ahi Evran University, Arts and Science Faculty, Department of Molecular Biology and Genetics, Kırşehir, Türkiye, [5]University of Health Sciences, Faculty of Pharmacy, Division of Biochemistry, Istanbul, Türkiye, [6]Recep Tayyip Erdogan University, Faculty of Medicine, Division of Biochemistry, Rize, Türkiye.

HPLC Chromatograms of Metabolite Analysis

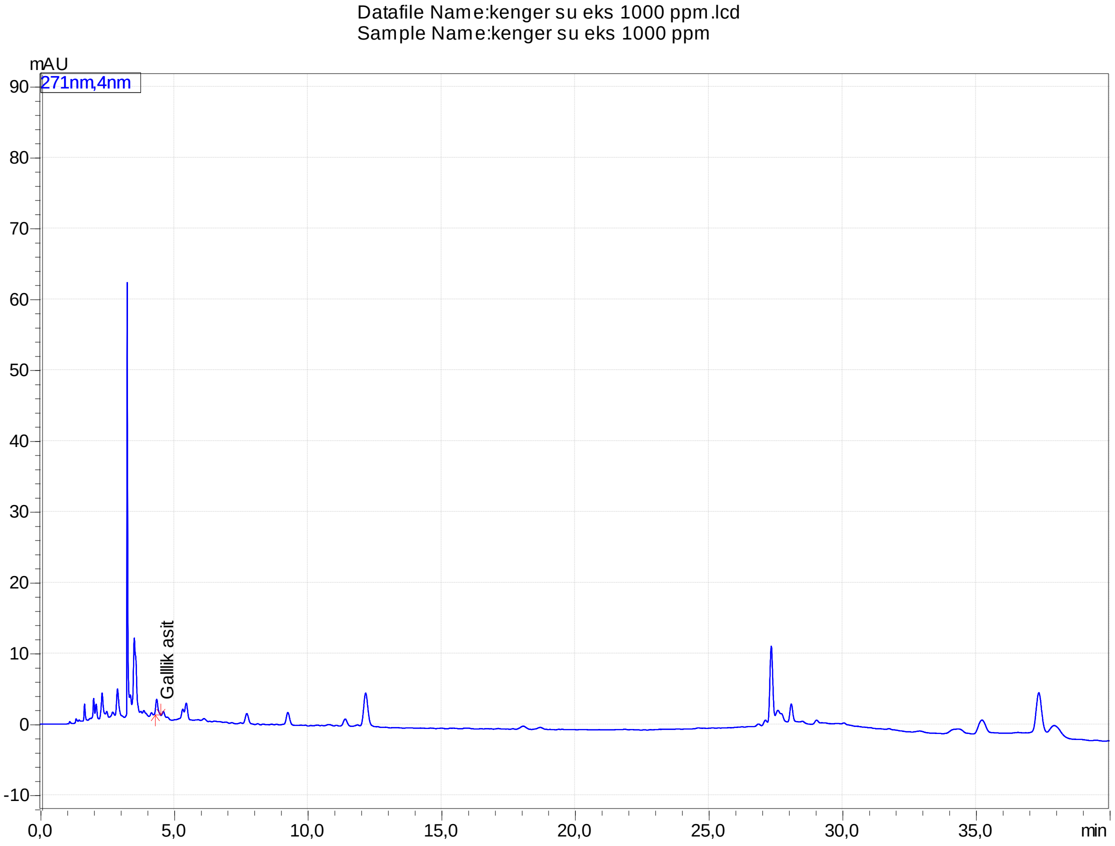

Figure S1: Gallic acid metabolite analysis

Peak#	Ret. Time	Conc.	Unit	Name	S/N
**1**	4,362	0,372	mg/L	Gallic acid	1,33
**Total**		0,372			

Table S1: Gallic acid peak properties

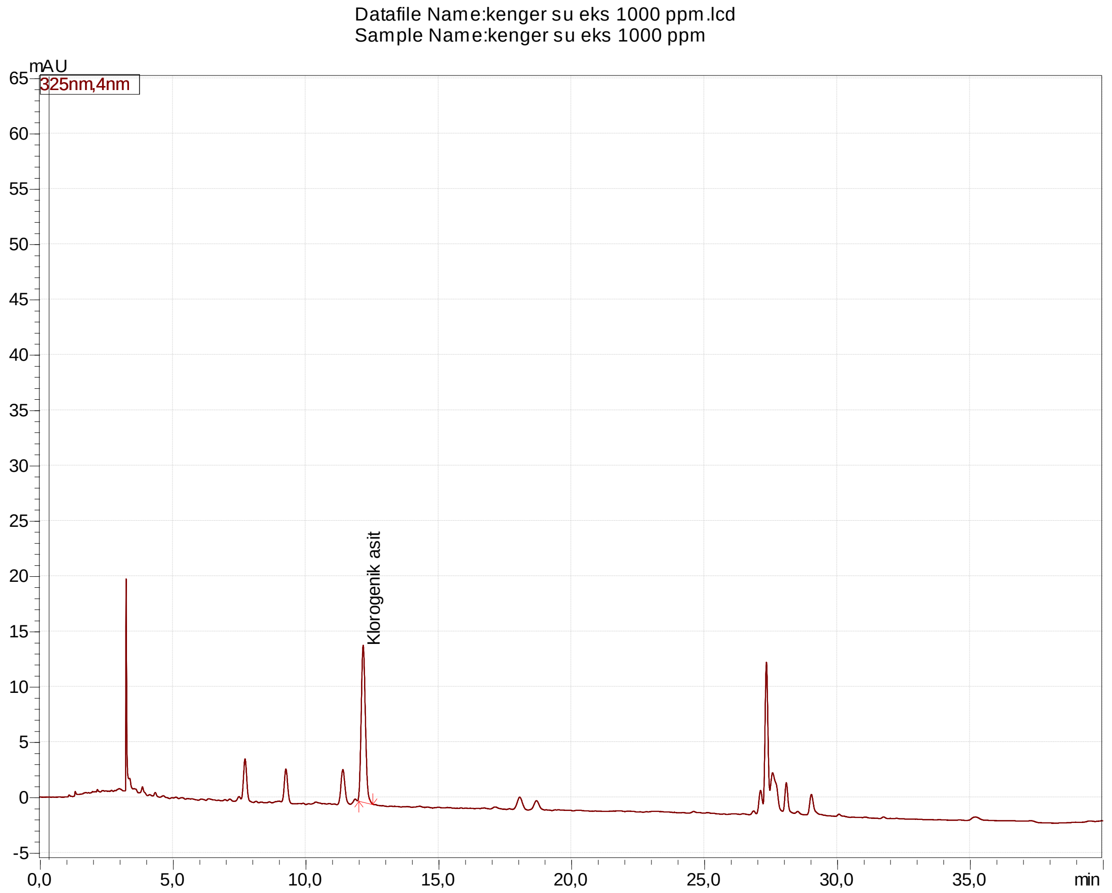

Figure S2: Clorogenic acid metabolite analysis

Peak#	Ret. Time	Conc.	Unit	Name	S/N
1	12,180	4,638	mg/L	Clorogenic acid	18,63
Total		4,638			

Table S2: Clorogenic acid peak properties

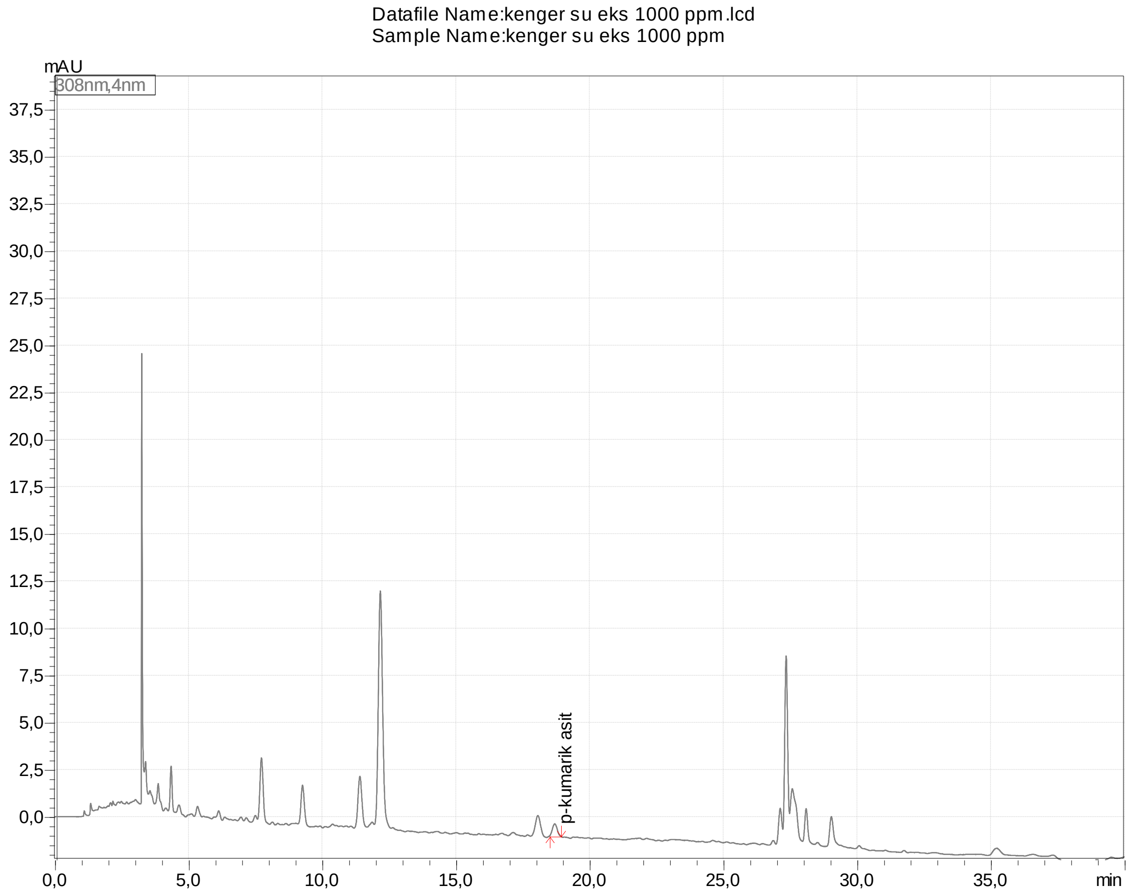

Figure S3: p-coumaric acid metabolite analysis

Peak#	Ret. Time	Conc.	Unit	Name	S/N
1	18,704	0,100	mg/L	p-coumaric acid	0,69
Total		0,100			

Table S3: p-coumaric acid peak properties

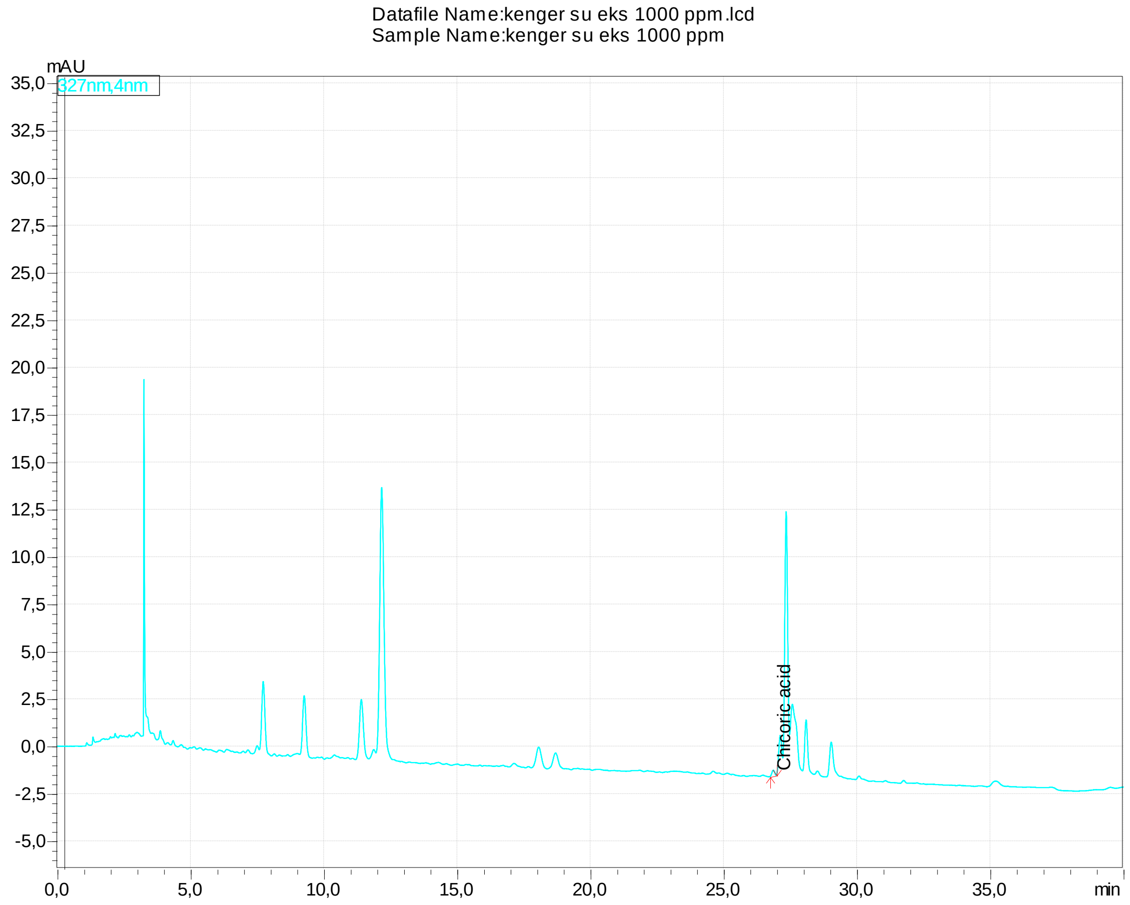

Figure S4: Chicoric acid metabolite analysis

Peak#	Ret. Time	Conc.	Unit	Name	S/N
1	26,875	0,185	mg/L	Chicoric acid	0,31
Total		0,185			

Table S4: Chicoric acid peak properties

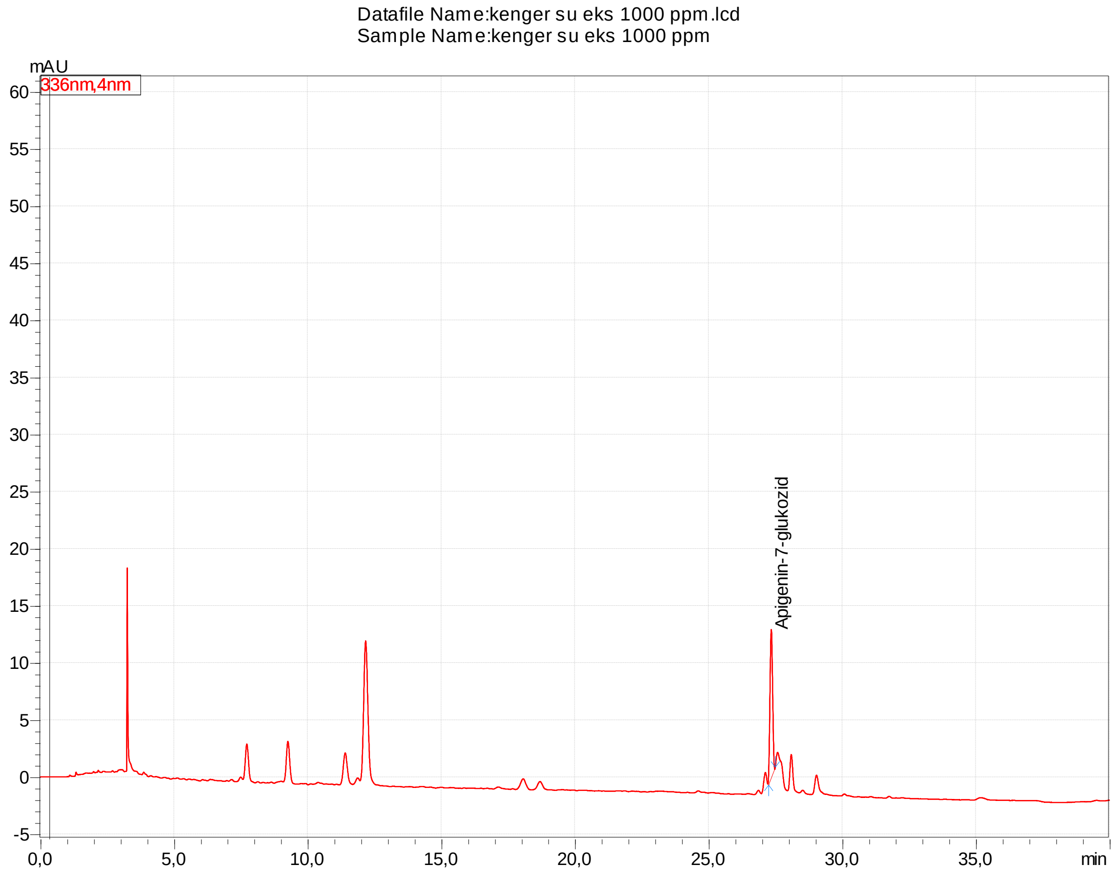

Figure S5: Apigenin-7-glucoside metabolite analysis

Peak#	Ret. Time	Conc.	Unit	Name	S/N
**1**	27,357	1,886	mg/L	Apigenin-7-glucoside	17,17
**Total**		1,886			

Table S5: Apigenin-7-glucoside peak properties

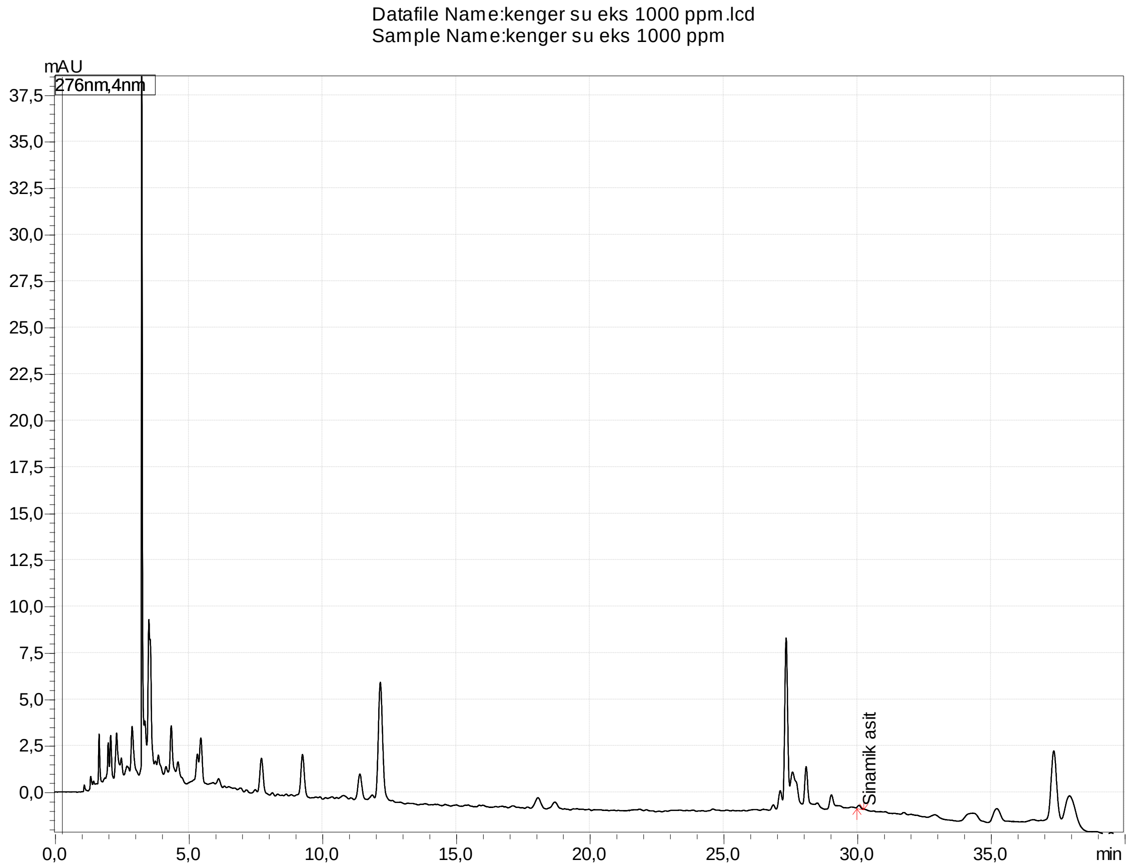

Figure S6: Cinnamic acid metabolite analysis

Peak#	Ret. Time	Conc.	Unit	Name	S/N
**1**	30,074	0,012	mg/L	Cinnamic acid	0,11
**Total**		0,012			

Table S6: Cinnamic acid peak properties

Overlay of standard chromatogram and sample chromatogram (at 254 nm)

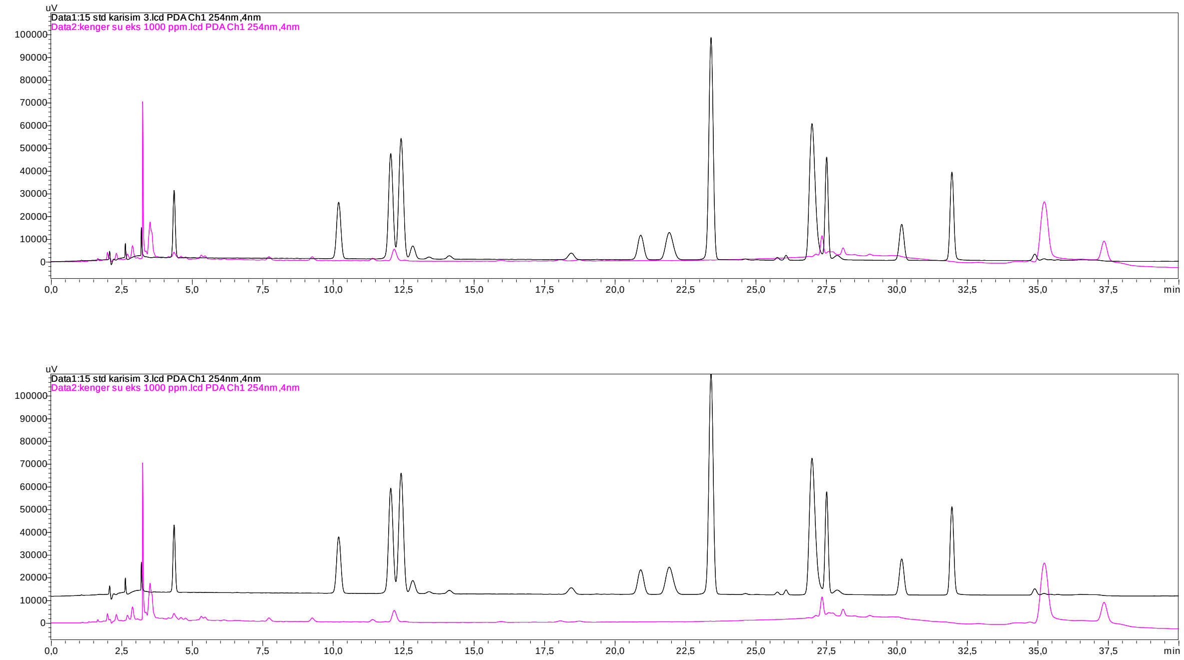

Figure S7: Overlay of standard chromatogram and sample chromatogram

ID#	Name	Ret. Time	Conc.	Unit	Channel	S/N
**1**	Gallic acid	4,362	0,372	mg/L	Ch2 271nm	1,33
**2**	4-hidroxybenzoic acid	No peak is detected.	0,000	mg/L	Ch1 254nm	--
**3**	Chlorogenic acid	12,180	4,638	mg/L	Ch3 325nm	18,63
**4**	Vanilic acid	No peak is detected.	0,000	mg/L	Ch4 260nm	-
**5**	Cafeic acid	No peak is detected.	0,000	mg/L	Ch5 248nm	-
**6**	Epicatechin	No peak is detected.	0,000	mg/L	Ch6 277nm	-
**7**	p-Coumaric acid	18,704	0,100	mg/L	Ch7 308nm	0,69
**8**	Ferulic acid	No peak is detected.	0,000	mg/L	Ch8 322nm	-

**9**	Salicylic acid	No peak is detected.	0,000	mg/L	Ch9 235nm	-
**10**	Rutin hydrate	No peak is detected.	0,000	mg/L	Ch1 254nm	-
**11**	Chicoric acid	26,875	0,185	mg/L	Ch10 327nm	0,31
**12**	Apigenin-7-glucoside	27,357	1,886	mg/L	Ch11 336nm	17,17
**13**	Cinnamic acid	30,074	0,012	mg/L	Ch12 276nm	0,11
**14**	Quercetin	No peak is detected.	0,000	mg/L	Ch1 254nm	--
**15**	Naringenin	No peak is detected.	0,000	mg/L	Ch13 288nm	-

Table S7: Metabolic content of *G. tournefortii*.
